# A systematic review of circulating IP-10/CXCL10 in patients with *Plasmodium* infections in relation to disease severity

**DOI:** 10.1038/s41598-024-82712-0

**Published:** 2024-12-30

**Authors:** Manas Kotepui, Aongart Mahittikorn, Frederick Ramirez Masangkay, Kwuntida Uthaisar Kotepui

**Affiliations:** 1 Medical Technology Program, Faculty of Science, Nakhon Phanom University, Nakhon Phanom, Thailand; 2 Department of Protozoology, Faculty of Tropical Medicine, Mahidol University, Bangkok, 10400 Thailand; 3 Department of Medical Technology, Faculty of Pharmacy, University of Santo Tomas, 1008 Manila, Philippines

**Keywords:** *Plasmodium*, Malaria, IP-10, CXCL10, Systematic review, Biomarkers, Malaria

## Abstract

Interferon γ-induced protein 10 kDa (IP-10) or C–X–C motif chemokine 10 (CXCL10) is produced and secreted from specific leukocytes such as neutrophils, eosinophils, and monocytes, which play key roles in the immune response to *Plasmodium* infections. This systematic review aimed to collate and critically appraise the current evidence on IP-10 levels in malaria patients. It provided insights into its role in malaria pathogenesis and potential as a biomarker for *Plasmodium* infections and disease severity. The protocol for this systematic review was registered in PROSPERO (number CRD42024556087). A comprehensive literature search was conducted across multiple databases, including Embase, PubMed, Scopus, Ovid, ProQuest, and MEDLINE, to identify relevant studies examining the role of IP-10 in patients with *Plasmodium* infections. A narrative synthesis was applied to summarize key findings and to provide an overview of the relationship between IP-10/CXCL10 levels and *Plasmodium* infection and disease severity. A total of 1933 records were identified, and 26 studies were included in the synthesis. The studies collectively indicated that IP-10 levels are elevated in patients with *Plasmodium* infections compared to healthy or non-malarial controls. Most studies reported that increased IP-10 levels were associated with increased disease severity. However, a few studies found no significant difference or decreased levels in patients with severe *Plasmodium* infections compared to those with uncomplicated or mild malaria. Additionally, several studies indicated that IP-10 levels were elevated in cerebral malaria. The systematic review suggests that IP-10 is elevated in patients with *Plasmodium* infections. However, the variability in findings across different studies regarding the association between IP-10 and severe malaria, particularly cerebral malaria, highlights the need for further comprehensive studies. Addressing confounding factors will be crucial in future research to better understand the role of IP-10 in *Plasmodium* infections and the pathogenesis of severe disease.

**Supplementary Information:**

The online version contains supplementary material available at 10.1038/s41598-024-82712-0.

## Introduction

Malaria remains a significant global health issue, particularly in tropical and subtropical regions^1^. It is caused by *Plasmodium* parasites, with *P. falciparum* and *P. vivax* being the most common and clinically significant species^2^. The disease is transmitted to humans through the bites of infected *Anopheles* mosquitoes^2^. Symptoms of malaria can range from mild, including fever, chills, and headaches, to severe, such as cerebral malaria, severe anemia, and multi-organ failure^3^. Humans who have not previously been exposed to malaria almost always become ill upon their first encounter with the parasite, with young children being particularly susceptible^4^. This is due to the development of immunity in high or moderate transmission areas, which allows older children and adults to achieve near-complete protection from severe malaria and death^4,5^.

Chemokines are crucial for directing leukocyte movement, enabling their migration to lymphoid tissues and deployment to peripheral sites of infection and inflammation^6^. Interferon γ-induced protein 10 kDa (IP-10) or C–X–C motif chemokine 10 (CXCL10) is a chemokine induced by interferon-gamma (IFN-γ) and tumor necrosis factor-alpha (TNF-α)^7^. IP-10 is produced and secreted from specific leukocytes such as neutrophils, eosinophils, and monocytes. In addition, IP-10 is secreted from epithelial, endothelial, stromal cells, and keratinocytes^7^. IP-10 is activated by binding to a seven trans-membrane-spanning G protein-coupled receptor called CXCR3^8^. This binding promotes the chemotactic activity of CXCR3 + cells (macrophages, dendritic cells, NK cells, and activated T lymphocytes), inducing and regulating cell growth and proliferation, apoptosis, and angiogenesis^9^. IP-10 has been reported to be a biomarker of several types of infections, including viral, bacterial, and protozoan infections^9–12^.

The role of IP-10 in *Plasmodium* infections was demonstrated in mouse models and participants infected with *Plasmodium*. For example, increased IP-10 mRNA expression was observed in *P. berghei* ANKA-infected mice with experimental cerebral malaria^13,14^. In human participants, increased IP-10 levels were associated with the development of cerebral malaria and cerebral malaria with an increased risk of mortality^15–17^. A recent study revealed that the polymorphisms in the IP-10 gene promoter sequence were associated with increased IP-10 production, resulting in a higher risk of severity of cerebral malaria^18^. This systematic review aimed to collate and critically appraise the current evidence on IP-10 in malaria, providing insights into its role in malaria pathogenesis and potential as a biomarker for *Plasmodium* infections and disease severity.

## Methods

### Protocol registration and reporting guideline

The protocol for this systematic review was registered in PROSPERO (number CRD42024556087). The systematic review was conducted according to the Preferred Reporting Items for Systematic Reviews and Meta-Analyses (PRISMA) guidelines^19^.

### Search strategy

A comprehensive literature search was conducted across multiple databases, including Embase, PubMed, Scopus, Ovid, ProQuest, and MEDLINE, to identify relevant studies examining the role of IP-10 in patients with *Plasmodium* infections. Searches were conducted for studies published up to May 2024. The search terms in combination with Boolean operators used included; (“interferon gamma-induced protein-10” OR “IP-10” OR “CXCL10” OR “Interferon-Inducible Protein 10” OR “Interferon Inducible Protein 10” OR “Small Inducible Cytokine B10” OR “IFN-gamma-Inducible Protein” OR “CXCL10 Chemokine” OR “gamma IP-10 Protein” OR “gamma IP 10 Protein” OR “Chemokine (C-X-C Motif) Ligand 10” AND (malaria OR *Plasmodium* OR “*Plasmodium* Infection“ OR “Remittent Fever“ OR “Marsh Fever“ OR Paludism) (Table S1). Additionally, reference lists of included studies were manually searched to identify further eligible studies. Google Scholar was also used to identify relevant studies not indexed in the main databases. The search included articles published up to the date of the review.

### Eligibility criteria

Studies were included if they met the following criteria: (i) Original research articles reporting on IP-10 levels in human subjects with confirmed *Plasmodium* infection, (ii) Studies comparing IP-10 levels between malaria patients and non-malarial controls, or among patients with different severities of malaria, (iii) Articles published in English. Exclusion criteria were: (i) Studies not involving human subjects, (ii) Reviews, meta-analyses, case reports, and conference abstracts, and (iii) Studies lacking relevant data on IP-10 levels.

### Study selection and data extraction

Two independent authors (MK and AM) screened titles and abstracts to identify potentially eligible studies. Full-text articles were retrieved and assessed for eligibility. Discrepancies were resolved by consensus or consulting a third reviewer (KUK). Data were extracted using a standardized data extraction form, which included study characteristics (author, year, country, and study design), participant characteristics (age, gender, and health status), *Plasmodium* species, methods for measuring IP-10, and main findings related to IP-10 levels.

### Quality assessment and data synthesis

The quality of the included studies was assessed using the Joanna Briggs Institute (JBI) critical appraisal checklists appropriate for each study design (case-control, cohort, and cross-sectional studies)^20^. The checklists evaluated aspects such as sample inclusion criteria, measurement validity and reliability, identification and management of confounding factors, and appropriateness of statistical analyses. A narrative synthesis^21^ was applied to summarize key findings and to provide an overview of the relationship between IP-10 levels and *Plasmodium* infection, disease severity, and clinical outcomes.

## Results

### Search results

**Fig. 1:**
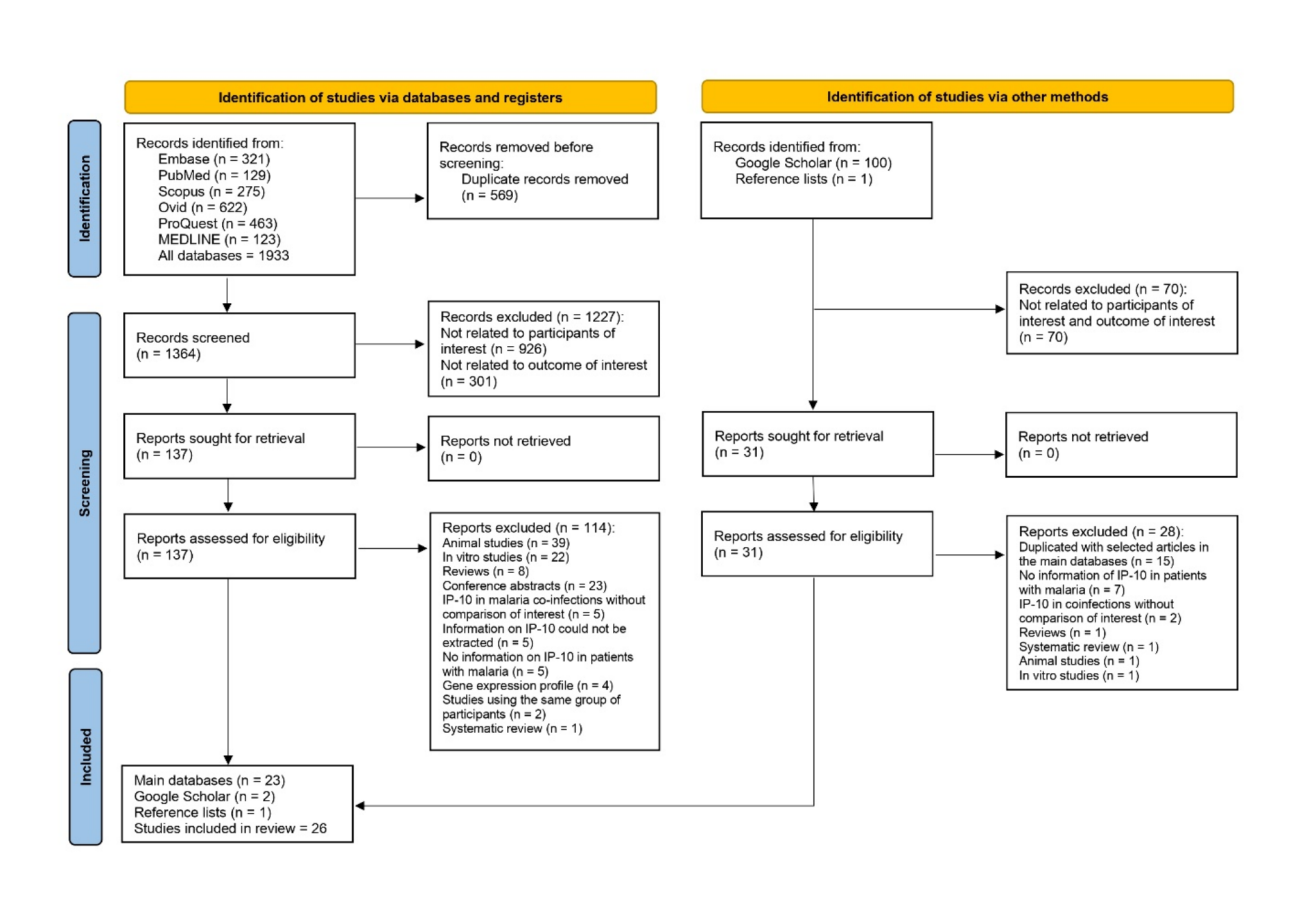
Study flow diagram.

Initially, 1933 records were identified from main databases; 100 additional records were sourced from Google Scholar and one article from the reference list. Starting with the 1933 records from the databases, after removing 569 duplicate records, 1364 records were screened, leading to the exclusion of 1227 records that were irrelevant to the participants or outcomes of interest. Subsequently, 137 reports were sought for full-text retrieval, all of which were successfully retrieved. During the eligibility assessment, 114 reports were excluded for reasons such as being animal studies, in vitro studies, reviews, conference abstracts, and lack of relevant information on IP-10 in malaria, resulting in 23 studies. Of the 100 records from Google Scholar and one from the reference list, 70 were excluded for not being related to participants or outcomes of interest, while all the remaining 31 records were retrieved. Of these, 28 were excluded for various reasons, and only 3 were included in the review. Finally, 26 studies were included in the review^15–18,22–43^, comprising 23 from the main databases^15–18,22–40^, two from Google Scholar^41,42^, and one from the reference list^43^ (Fig. 1).

### General characteristics of 26 included studies

An overview of the general characteristics of the 26 included studies on IP-10 levels in malaria patients is demonstrated in Table 1. Most of these studies (69.23%) were published between 2010 and 2019. Regarding study design, the majority (61.54%) were case-control studies. Geographically, most of the studies were conducted in Africa (53.84%), with a significant number in Ghana (15.38%). Regarding the *Plasmodium* species, most studies focused on *P. falciparum* (69.23%). The participant demographics varied, but most studies involved children (34.62%) or adults (30.77%). Diagnostic methods for malaria predominantly used the microscopic method, either alone (50%) or combined with PCR or RDT. For IP-10 level assays, the majority (65.38%) utilized bead-based immunoassays, and nearly all studies (96.15%) analyzed plasma samples. Detailed characteristics of the included studies are listed in Table 2.

**Table 1:** General characteristics of included studies.

Characteristics	*n*. (26 studies)	%
Publication year		
2000–2009	2	7.69
2010–2019	18	69.23
2020–2023	6	23.08
Study designs		
Case-control study	16	61.54
Cross-sectional study	5	19.23
Cohort study	5	19.23
Study areas		
Asia	7	26.92
India	6	23.08
Pakistan	1	3.85
Africa	14	53.84
Ghana	4	15.38
Mali	3	11.54
Kenya	1	3.85
Malawi	1	3.85
Benin	1	3.85
Mozambique	1	3.85
Tanzania	1	3.85
Togo	1	3.85
Uganda	1	3.85
South America	4	15.38
Brazil	3	11.54
Colombia	1	3.85
Multi-countries	1	3.85
Brazil, Colombia, Guatemala, India, Papua New Guinea	1	3.85
*Plasmodium* species		
*P. falciparum*	18	69.23
*P. vivax*	6	23.08
*P. falciparum*, mixed *P. falciparum/P. vivax* infections, mixed *P. falciparum/P. malariae*	1	3.85
Not specified species	1	3.85
Participants		
Children	9	34.62
Adults	8	30.77
Children and adults	6	23.08
Pregnant women	3	11.54
Methods for malaria detection		
Microscopic method	13	50.00
Microscopic method/PCR	5	19.23
Microscopic method/RDT	4	15.38
Microscopic method/RDT/PCR	4	15.38
Assays for IP-10		
Bead-based immunoassay	17	65.38
Enzyme Immunoassay	9	34.62
Blood samples		
Plasma	25	96.15
Serum	1	3.85

PCR, polymerase chain reaction; RDT, rapid diagnostic test.

**Table 2:** Detailed characteristics of included studies.

No.	Authors	Study location	*Plasmodium* spp.	Age range of participants	Method for *Plasmodium* detection	Method for IP-10 detection	Samples	Findings of individual study
1.	Acero et al., 2022^22^	Colombia	*P. vivax*	Children and adults	Microscopic method/PCR	Bead-based immunoassay	Plasma	- A significantly higher IP-10 was observed in non-severe malaria patients compared to healthy controls. - A significantly higher IP-10 was observed in severe compared to non-severe malaria patients
2.	Armah et al., 2007^15^	Ghana	*P. falciparum*	Children	Microscopic method	Bead-based immunoassay	Serum CSF	IP-10 in serum; - A significantly higher IP-10 was observed in cerebral malaria patients compared to non-malaria deaths. - A significantly higher IP-10 was observed in cerebral malaria compared to severe malarial anemia. - IP-10 was lower in severe malarial anemia compared to non-malaria deaths (the difference was not statistically significant). IP-10 in CSF; - A significantly higher IP-10 was observed in cerebral malaria compared to non-malaria deaths. - A significantly higher IP-10 was observed in cerebral malaria compared to severe malarial anemia. - IP-10 was higher in severe malarial anemia compared to non-malaria deaths (the difference was not statistically significant).
3.	Berg et al., 2014^23^	Mozambique	*P. falciparum*,* mixed P. falciparum/P. vivax infections*,* mixed P. falciparum/P. malariae* infections	Adults	Microscopic method/RDT/PCR	Bead-based immunoassay	Plasma	- A significantly higher IP-10 was observed in malaria patients (uncomplicated and severe malaria) compared to healthy controls. - A significantly higher IP-10 in severe malaria compared with uncomplicated malaria patients.
4.	Boström et al., 2012^24^	Mali	*P. falciparum*	Children	Microscopic method	Bead-based immunoassay	Plasma	- A significantly higher IP-10 was observed in *Plasmodium*-infected patients compared to healthy controls (Dogon). - IP-10 was higher in *Plasmodium*-infected patients compared to healthy controls (Fulani) (the difference was not statistically significant).
5.	Boström et al., 2012^25^	Tanzania	*P. falciparum*	Pregnant women	Microscopic method/RDT	Bead-based immunoassay	Plasma	- A significantly higher IP-10 was observed in *Plasmodium*-infected women, irrespective of gravidity, compared to uninfected women.
6.	Cabantous et al., 2020^26^	Mali	*P. falciparum*	Children	Microscopic method	Enzyme immunoassay	Plasma	- A significantly lower IP-10 was observed in cerebral malaria compared to uncomplicated malaria patients. - Cerebral malaria who died from malaria tended to have higher IP-10 compared to those who recovered.
7.	Conroy et al., 2010^27^	Malawi	*P. falciparum*	Children	Microscopic method	Enzyme immunoassay	Plasma	- IP-10 was higher in cerebral malaria with retinopathy compared to those without retinopathy (the difference was not statistically significant). - IP-10 was higher in cerebral malaria with retinopathy compared to uncomplicated malaria patients (the difference was not statistically significant). - IP-10 was higher in cerebral malaria with retinopathy compared to children with fever and decreased consciousness due to other causes (the difference was not statistically significant).
8.	Cruz et al., 2019^41^	Brazil	*P. vivax*	Adults	Microscopic method/PCR	Bead-based immunoassay	Plasma	- A significantly lower IP-10 was observed in vivax patients compared to healthy controls. - A significantly lower IP-10 was observed in asymptomatic vivax malaria patients compared to healthy controls. - No significant increase in IP-10 was observed in symptomatic vivax patients compared to healthy controls.
9.	Dalko et al., 2015^28^	India	*P. falciparum*	Adults	Microscopic method/RDT/PCR	Bead-based immunoassay	Plasma	- IP-10 was higher in cerebral malaria compared to mild malaria patients (the difference was not statistically significant). - IP-10 was higher in acute renal failure compared to mild malaria patients (the difference was not statistically significant).
10.	Dobaño et al., 2020^29^	Brazil, Colombia, Guatemala, India, Papua New Guinea	*P. vivax*	Pregnant women	Microscopic method/PCR	Bead-based immunoassay	Plasma	- A significantly higher IP-10 was observed in *P. vivax–*infected pregnant women compared to uninfected women.
11.	Erdman et al., 2011^30^	Uganda	*P. falciparum*	Children	Microscopic method	Enzyme immunoassay	Plasma	- IP-10 was higher in cerebral malaria compared to uncomplicated malaria patients (the difference was not statistically significant). - IP-10 was higher in cerebral malaria compared to severe malarial anemia (the difference was not statistically significant). - IP-10 was lower in severe malarial anemia compared to uncomplicated malaria patients (the difference was not statistically significant). - A significantly higher IP-10 was observed in children with severe malaria who died compared to those who survived *Plasmodium* infection.
12.	Frimpong et al., 2022^43^	Ghana	*P. falciparum*	Children	Microscopic method/RDT	Bead-based immunoassay	Plasma	- A significantly higher IP-10 was observed in children with malaria compared to febrile controls. - A significantly lower IP-10 was observed in children with sepsis compared to children with malaria. - A significantly higher IP-10 was observed in children with sepsis compared to febrile controls.
13.	Ghanchi et al., 2015^31^	Pakistan	*P. falciparum*	Adults	Microscopic method	Enzyme immunoassay	Plasma	- A significantly higher IP-10 was observed in uncomplicated malaria compared to non-malarial controls. - A significantly higher IP-10 was observed in severe malaria compared to non-malarial controls. - IP-10 was higher in severe malaria compared to uncomplicated malaria patients (the difference was not statistically significant).
14.	Herbert et al., 2015^32^	India (2008–2010)	*P. falciparum*	Children and adults	Microscopic method/RDT/PCR	Bead-based immunoassay	Plasma	- A significantly higher IP-10 was observed in severe malaria compared to non-malarial controls. - IP-10 was higher in severe malaria (cerebral and severe non-cerebral malaria) compared to mild malaria patients (the difference was not statistically significant). - IP-10 can distinguish cerebral malaria patients from patients with multiple organ dysfunction.
15.	Hojo-Souza et al., 2017^33^	Brazil	*P. vivax*	Adults	Microscopic method/PCR	Bead-based immunoassay	Plasma	- A significantly higher IP-10 was observed in uncomplicated malaria patients compared to non-malarial controls. - A significantly higher IP-10 was observed in uncomplicated malaria patients compared to healthy controls. - A significantly lower IP-10 was observed in *P. vivax*-treated group compared to uncomplicated malaria patients
16.	Ibitokou et al., 2014^34^	Benin	*P. falciparum*	Pregnant women	Microscopic method/RDT/PCR	Bead-based immunoassay	Plasma	- A significantly higher IP-10 was observed in pregnant women with malaria compared to uninfected controls (at inclusion and delivery).
17.	Jain et al., 2008^16^	India	*P. falciparum*	Children and adults	Microscopic method	Bead-based immunoassay	Plasma	- IP-10 was higher in mild malaria compared to healthy controls (the difference was not statistically significant). - A significantly higher IP-10 was observed in cerebral malaria (survivors or non-survivors) compared to mild malaria patients. - A significantly higher IP-10 was observed in cerebral malaria (survivors or non-survivors) compared to healthy controls. - IP-10 was higher in cerebral malaria non-survivors compared to cerebral malaria survivors (the difference was not statistically significant).
18.	Jain et al., 2010^35^	India	*P. vivax*	Children and adults	Microscopic method	Enzyme immunoassay	Plasma	- A significantly higher IP-10 was observed in malaria patients compared to healthy controls.
19.	Lekpor et al., 2022^36^	Ghana	Not specified	Adults	Microscopic method/RDT	Enzyme immunoassay	Plasma saliva	IP-10 in plasma; - A significantly higher IP-10 was observed in malaria patients compared to non-malarial controls. IP-10 in saliva; - A significantly higher IP-10 was observed in malaria patients compared to non-malarial controls.
20.	Lopera-Mesa et al., 2012^37^	Mali	*P. falciparum*	Children	Microscopic method	Bead-based immunoassay	Plasma	- A significantly higher IP-10 was observed in severe malaria compared to uncomplicated malaria. - A significantly higher IP-10 was observed in cerebral malaria compared to non-cerebral severe malaria patients.
21.	Mendonça et al., 2013^42^	Brazil	*P. vivax*	Children and adults	Microscopic method/PCR	Bead-based immunoassay	Plasma	- IP-10 levels were highest in severe malaria patients who died, followed by severe malaria survivors, symptomatic malaria patients, and asymptomatic malaria patients.
22.	Obeng-Aboagye et al., 2023^38^	Ghana	*P. falciparum*	Children	Microscopic method	Bead-based immunoassay	Plasma	- IP-10 was higher in severe malaria compared to uncomplicated malaria patients (the difference was not statistically significant). - A significantly higher IP-10 was observed in severe malaria compared to non-malarial controls
23.	Ong’echa et al., 2011^39^	Kenya	*P. falciparum*	Children	Microscopic method	Bead-based immunoassay	Plasma	- A significantly higher IP-10 was observed in non-severe anemia patients compared to uncomplicated malaria patients. - IP-10 was higher in severe malarial anemia compared to uncomplicated malaria patients (the difference was not statistically significant).
24.	Wangala et al., 2015^40^	Togo	*P. falciparum*	Children and adults	Microscopic method/RDT	Enzyme immunoassay	Plasma	- A significantly higher IP-10 was observed in malaria patients compared to non-malarial controls. - A significantly higher IP-10 was observed in severe malaria compared to uncomplicated malaria patients.
25.	Wilson et al., 2013^18^	India	*P. falciparum*	Adults	Microscopic method	Enzyme immunoassay	Plasma	- A significantly higher IP-10 was observed in cerebral malaria compared to non-cerebral malaria patients. - Both males and females in the cerebral malaria group had significantly higher IP-10 levels than their corresponding gender in the non-cerebral malaria group. - IP-10 levels were higher in males compared to females within the non-cerebral malaria group (though the difference was not statistically significant). - A significantly higher IP-10 was observed in males compared to females within the cerebral malaria group.
26.	Wilson et al., 2011^17^	India	*P. falciparum*	Adults	Microscopic method	Enzyme immunoassay	Plasma	- IP-10 was higher in uncomplicated malaria compared to healthy controls (the difference was not statistically significant). - A significantly higher IP-10 was observed in cerebral malaria non-survivors compared to healthy controls. - A significantly higher IP-10 was observed in cerebral malaria (survivors and non-survivors) compared to uncomplicated malaria patients. - A significantly higher IP-10 was observed in cerebral malaria non-survivors compared to cerebral malaria survivors.

RDT, rapid diagnostic test; PCR, polymerase chain reaction.

### IP-10 levels in malaria patients compared to non-malarial controls

Eighteen studies compared IP-10 levels between malaria patients and non-malarial controls^16,17,22–25,29,31–38,40,41,43^. The studies collectively indicated that IP-10 levels are generally elevated in patients with malaria compared to healthy or non-malarial controls^22,23,25,29,32–38,40,43^. However, some studies found no significant difference in IP-10 levels between uncomplicated malaria and healthy controls^16,17,24,31^. Only one study (Cruz et al., 2019) reported lower IP-10 levels in *Plasmodium*-infected individuals compared to healthy controls^41^.

For the role of IP-10 in *P. falciparum* infections, Armah et al. showed that IP-10 levels were higher in severe malarial anemia compared to non-malarial deaths, although the difference was not statistically significant in both serum and cerebrospinal fluid samples (CSF)^15^. Boström et al. revealed a significantly higher IP-10 in *Plasmodium*-infected patients compared to healthy controls (Dogon), but while IP-10 levels were also higher in *Plasmodium*-infected patients compared to healthy controls (Fulani), the difference was not statistically significant. Frimpong et al. observed a significantly higher IP-10 in children with malaria compared to febrile controls, a significantly lower IP-10 in children with sepsis compared to those with malaria, and a significantly higher IP-10 in children with sepsis compared to febrile controls^43^. Ghanchi et al. found significantly higher IP-10 levels in severe malaria compared to non-malarial controls and significantly higher IP-10 in uncomplicated malaria compared to non-malarial controls. Herbert et al. showed a significantly higher IP-10 in severe malaria compared to non-malarial controls^31^. Jain et al. reported that IP-10 levels were higher in mild malaria compared to healthy controls, though the difference was not statistically significant^16^. Obeng‑Aboagye et al. observed a significantly higher IP-10 in severe malaria compared to non-malarial controls^38^. Wangala et al. found a significantly higher IP-10 in malaria patients compared to non-malarial controls^40^.

### IP-10 levels in *P. vivax* malaria

For the role of IP-10 in *P. vivax* infections, Cruz et al. observed significantly lower IP-10 levels in *P. vivax* infections compared to healthy controls. They also reported significantly lower IP-10 levels in asymptomatic *P. vivax* infections compared to healthy controls and no significant increase in IP-10 was observed in symptomatic vivax patients compared to healthy controls^41^. Dobaño et al. found significantly higher IP-10 levels in *P. vivax*–infected pregnant women compared to uninfected women^29^. Hojo-Souza et al. reported significantly higher IP-10 levels in uncomplicated *P. vivax* infections compared to non-malarial controls and healthy controls. Additionally, they observed significantly lower IP-10 levels in the *P. vivax*-treated group compared to *P. vivax* uncomplicated patients^33^. Jain et al. found significantly higher IP-10 levels in *P. vivax* infections compared to healthy controls^35^. Mendonça et al. observed that IP-10 levels were highest in severe *P. vivax* malaria patients who died, followed by severe malaria survivors, symptomatic malaria patients, and asymptomatic malaria patients^42^.

### IP-10 levels in patients with severe malaria compared to those with non-severe malaria

The studies on IP-10 levels in malaria patients show mixed results^16,17,22,23,26–28,30–32,38–40,42^. Six studies reported that increased IP-10 levels were associated with increased disease severity^16,17,22,23,40,42^. Wilson et al.^17^ and Jain et al.^16^ reported significantly higher IP-10 levels in adults and children with cerebral malaria compared to uncomplicated or mild malaria. Similarly, Acero et al.^22^ and Wangala et al.^40^ observed higher IP-10 levels in severe cases compared to non-severe or uncomplicated cases. Berg et al. supported these findings with elevated IP-10 in severe malaria compared to uncomplicated cases^23^. Mendonça et al. noted increased IP-10 levels with disease severity in *P. vivax* infections in which IP-10 levels were highest in severe *P. vivax* malaria patients compared to symptomatic malaria patients^42^. Interestingly, Ong’echa et al. found significantly higher IP-10 levels in children with non-severe malarial anemia compared to those with uncomplicated malaria, but no significant difference between severe malarial anemia and uncomplicated malaria^39^.

Conversely, Dalko et al.^28^ and Erdman et al.^30^ observed no significant differences in IP-10 levels between cerebral or severe malaria and mild or uncomplicated malaria in adults and children, respectively. However, Cabantous et al. observed lower IP-10 levels in children with cerebral malaria compared to uncomplicated malaria^26^. Contrarily, Conroy et al.^27^, Herbert et al.^32^, Ghanchi et al.^31^, and Obeng‑Aboagye et al.^38^ found no significant differences in IP-10 levels between severe and mild or uncomplicated malaria.

### IP-10 levels in fatal cerebral malaria vs. non-fatal cerebral malaria vs. uncomplicated malaria

Jain et al. reported a significantly higher level of IP-10 in cerebral malaria patients (both survivors and non-survivors) compared to those with mild malaria. They also observed that IP-10 levels were higher in cerebral malaria non-survivors compared to survivors, though this difference was not statistically significant^16^. Erdman et al. found significantly higher IP-10 levels in children with severe malaria who died compared to those who survived^30^. Wilson et al. observed significantly higher IP-10 levels in cerebral malaria non-survivors compared to survivors^17^. Additionally, they reported significantly higher IP-10 levels in cerebral malaria patients (both survivors and non-survivors) compared to those with uncomplicated malaria^17^. Mendonça et al. observed that IP-10 levels were highest in severe *P. vivax* malaria patients who died, followed by severe malaria survivors, and symptomatic malaria patients^42^. Cabantous et al. observed that patients with cerebral malaria who died from the complication tended to have higher IP-10 levels compared to those who recovered^26^.

### IP-10 levels in cerebral malaria and severe non-cerebral malaria

Armah et al. observed significantly higher IP-10 levels in cerebral malaria compared to severe malarial anemia in both serum and CSF samples^15^. Erdman et al. found that IP-10 levels were higher in cerebral malaria compared to severe malarial anemia, although the difference was not statistically significant^30^. Lopera-Mesa et al. also showed significantly higher IP-10 levels in cerebral malaria compared to non-cerebral severe malaria patients^37^. Wilson et al. reported significantly higher IP-10 levels in cerebral malaria compared to those with non-cerebral malaria patients^18^. Herbert et al. also observed that IP-10 can distinguish cerebral malaria patients from malaria patients with multiple organ dysfunction^32^. IP-10 levels were higher in cerebral malaria with retinopathy compared to those without retinopathy, uncomplicated malaria, and children with fever and decreased consciousness due to other causes; however, none of these differences were statistically significant^27^.

### IP-10 levels in pregnant women with *Plasmodium* infections

For the role of IP-10 in pregnant women with *Plasmodium* infections, Boström et al. showed that significantly higher IP-10 levels were observed in *Plasmodium*-infected women, irrespective of gravidity, compared to uninfected women^25^. Dobaño et al. revealed significantly higher IP-10 levels in *P. vivax*–infected pregnant women compared to uninfected women^29^. Ibitokou et al. found significantly higher IP-10 levels in pregnant women with malaria compared to uninfected controls, both at inclusion and delivery^34^.

### Risk of bias of included studies

The risk of bias assessment using the JBI critical appraisal checklist is shown in Table S2. Five cross-sectional studies clearly defined the inclusion criteria, described study subjects and settings in detail, measured exposure validly and reliably, used objective and standard criteria for condition measurement, and performed the appropriate statistical analyses. Three studies identified confounding factors and applied strategies to address them^18,23,24^. However, two had unclear identification of confounding factors and did not state strategies to manage them^26,28^. The case-control studies clearly defined the inclusion criteria, used appropriate matching for cases and controls, applied the same criteria for case identification, measured exposure and outcomes in a standard, valid, and reliable way, and used the appropriate statistical analyses. However, some studies had unclear identification of confounding factors and did not state strategies to address them^31,41^. Five cohort studies ensured that the two groups were similar and recruited from the same population and that exposures were measured consistently and reliably. Confounding factors were identified, and strategies to address them were stated in every study. The outcomes were measured validly and reliably. Most studies did not fully address or explore insufficient follow-up time and incomplete follow-up. Three studies had notable issues with follow-up time and completeness^22,25,37^.

## Discussion

The synthesis of IP-10 levels in malaria patients versus non-malarial controls across 19 studies revealed a consistent trend of elevated IP-10 levels in malaria patients. This finding is robust across various age groups, geographic locations, and *Plasmodium* species, specifically *P. falciparum* and *P. vivax*^15,22,23,25,29,32–38,40,43^. However, it is essential to note that several studies indicated no significant difference in IP-10 levels between patients with uncomplicated malaria and healthy controls^16,17,24,31^. This may suggest that the elevation in IP-10 is more closely associated with severe malaria manifestations rather than mild or uncomplicated cases. Interestingly, Cruz et al.^41^ found lower IP-10 levels in *P. vivax* patients compared to healthy controls, a finding that contrasts with the general trend.

IP-10, produced in response to IFN-γ, attracts immune cells to the site of inflammation^44^. Changes in IP-10 expression levels have been linked to inflammatory conditions, including infectious diseases, immune dysfunction, and tumor development^9^. In a mouse model, IP-10 was first reported as a host-protective factor in murine experimental malaria^45^. Additionally, IP-10 has been associated with cerebral malaria in murine models by activating CD8 + T cells, which develop cerebral malaria in conjunction with the chemokine receptor CXCR3^13^.

For the evidence of IP-10 in different populations, a study revealed that IP-10 was increased in infected Dogon but not Fulani children compared to healthy controls, proposing that Fulani children may have some high baseline levels of IP-10 in circulation compared to Dogon children^24^. In pregnant women, IP-10 was significantly elevated at the time of infection and also at delivery, indicating that IP-10 levels were increased irrespective of gravidity^25^. Therefore, IP-10 is associated with *Plasmodium* infection in the placenta. IP-10 is one of the chemokines that attract monocyte and macrophage populations to the sites of infection^46^, indicating a pivotal role in the immunopathogenesis of IP-10 in pregnancy.

The data on IP-10 levels in patients with severe versus non-severe malaria demonstrated mixed results. Several studies reported significantly higher IP-10 levels in severe cases compared to mild or uncomplicated malaria. For instance, Wilson et al.^17^ and Jain et al.^16^ observed elevated IP-10 levels in cerebral malaria compared to uncomplicated malaria. Similarly, Acero et al.^22^, Wangala et al.^40^, and Berg et al.^23^ reported higher IP-10 levels in severe cases. In contrast, studies by Dalko et al.^28^ and Erdman et al.^30^ found no significant differences between severe and uncomplicated malaria, indicating variability in IP-10 response across different populations and study conditions. Cabantous et al.^26^ noted lower IP-10 levels in children with cerebral malaria compared to uncomplicated malaria, further complicating the relationship between IP-10 levels and disease severity. This variability underscores the complexity of IP-10’s role in malaria pathogenesis and suggests that its utility as a biomarker may depend on specific clinical and demographic contexts. A strong correlation between IP-10 and the severity of *Plasmodium* infections was observed in *P. falciparum* infections^23^.

For the role of IP-10 in cerebral malaria, a study reported that a significant elevation of serum and CSF levels of IP-10 is associated with cerebral malaria mortality, suggesting that IP-10 plays a substantial role in the immunopathology of cerebral malaria^15^. The reason for the elevation of IP-10 in cerebral malaria is that IP-10 is produced and expressed in astrocyte cells in the brain parenchyma in response to *Plasmodium* infections, as previously demonstrated^47^. Therefore, differences in the inflammatory response to *Plasmodium* infections depend on the type of cells that produce the IP-10 inflammatory mediator (brain cells or other cells)^15^, which might contribute to the varying results observed in the studies included in the systematic review conducted in different areas. In addition, IP-10 in combination with TNF-α may induce apoptosis of endothelial cells, leading to blood-brain barrier breakdown^15^. Cabantous et al.^26^ reported that the expression of IP-10 was higher in patients with uncomplicated malaria than those with cerebral malaria who recovered, suggesting that IP-10 may have a protective effect against the severe disease caused by *P. falciparum* infections. Nevertheless, excessive IP-10 may lead to vascular injury, leading to a blood-brain barrier breakdown. This breakdown could result in the accumulation of leukocytes, which induce local hyperinflammation and contribute to a lethal neuropathological syndrome^26^.

For the role of IP-10 as a prognostic marker for malaria, a previous study suggested that IP-10 level was regulated by hemopexin^18^ and that IP-10 alone is not associated with the outcome or a predictor of cerebral malaria^16,32^. Instead, IP-10 is proposed to be a vital component of the complex immune network involving heme and hemopexin in the pathophysiology of cerebral malaria^28^. A study revealed that IP-10 levels were highest among non-survivors of cerebral malaria, indicating that IP-10 was associated with mortality^16^. This finding was supported by a study in an infected mouse model, where IP-10 and the macrophage-induced gene (MIG) were found to play a role in attracting immune cells such as CD8 + T and NK cells to the brain, which may be related to death^13^.

Beyond the comparison between malaria and non-malarial controls, additional findings provide further insights into the role of IP-10 in malaria. For example, Jain et al.^16^ found no difference in IP-10 levels between cerebral malaria survivors and non-survivors, while Wilson et al.^18^ reported higher levels in survivors. This discrepancy suggests that IP-10 may not be a straightforward prognostic marker. Cruz et al.^41^ observed lower IP-10 levels in asymptomatic *P. vivax* patients compared to healthy controls but no significant alteration in symptomatic patients, indicating that the symptomatic state of the infection might modulate IP-10 expression. Studies by Lopera-Mesa et al.^37^ and Armah et al.^15^ found significantly higher IP-10 levels in cerebral malaria compared to non-cerebral severe malaria and severe malarial anemia, suggesting a potential role for IP-10 in the pathogenesis of cerebral malaria. Conversely, studies by Herbert et al.^32^ and Conroy et al.^27^ suggest that IP-10 might help distinguish cerebral malaria from other severe forms like multiple organ dysfunction but not from retinopathy-positive or negative cases. Lastly, IP-10 was found to differentiate children with clinical malaria from those with sepsis and other febrile conditions^43^.

An important theme noted from the systematic review is the variability in IP-10 levels across different populations, *Plasmodium* species, and malaria severities. While elevated IP-10 levels are generally observed in patients with severe and cerebral malaria, conflicting results exist due to differences in study design, population genetics, disease phase, and assay techniques. The role of IP-10 as both a protective and pathological immune mediator further complicates its potential as a biomarker. For example, while IP-10 is consistently elevated in severe *P. falciparum* malaria, its expression in *P. vivax* malaria appears less consistent, with a study even reporting lower IP-10 levels in symptomatic *P. vivax* patients^41^. IP-10’s involvement in pregnancy-associated malaria also suggests a role in placental pathology. Despite its promise as a biomarker, inconsistencies across studies indicate that elevated IP-10 may be more closely associated with severe malaria than uncomplicated cases. Future large-scale, multi-center studies are needed to standardize assays, investigate the mechanisms behind IP-10’s variable expression, and explore its clinical utility in identifying high-risk patients and guiding treatment strategies.

There are several limitations in this systematic review that should be acknowledged. First, the meta-analysis of IP-10 levels between individuals infected with *Plasmodium* and non-infected controls and the association between IP-10 levels and malaria severity could not be performed due to the limited number of studies reporting quantitative data. Additionally, inconsistent findings regarding the relationship between IP-10 levels and malaria severity present a challenge, with some studies showing clear associations and others reporting no significant differences. This variability may be attributed to several factors, including population differences in geographic location, age groups, and genetic backgrounds. Moreover, species-specific differences between *P. falciparum* and *P. vivax* infections contribute to the inconsistencies, with some studies reporting contrasting trends in IP-10 levels. Methodological differences, such as variations in study design, measurement techniques, and assay sensitivities, further confound the outcomes.

Other confounding factors, such as differences in immune status, co-infections, or underlying health conditions, were not always adequately controlled, complicating the interpretation of results. Additionally, insufficient follow-up time and incomplete follow-up in certain studies may have limited the ability to assess longitudinal changes in IP-10 levels and their association with malaria outcomes. The potential for bias arising from variability in study design, data collection, and analysis also poses a risk to the validity of the findings. Finally, differences in how malaria severity and outcomes were measured across studies contributed to the challenges in drawing definitive conclusions regarding the prognostic value of IP-10. Furthermore, the immune response that drives IP-10 production is influenced by several factors that could not be adequately stratified or controlled due to the small number of included studies in this review. These factors include malaria transmission intensity, the age of participants (e.g., children vs. adults), and physiological differences such as pregnancy status.

## Conclusion

The systematic review suggests that the IP-10 level is elevated in patients with *Plasmodium* infections. However, the variability in findings across different studies regarding the association between IP-10 and severe malaria, particularly cerebral malaria, highlights the need for further comprehensive studies. Addressing confounding factors will be crucial in future research to better understand the role of IP-10 levels in *Plasmodium* infections and the pathogenesis of severe disease.

## Electronic supplementary material

Below is the link to the electronic supplementary material.

Supplementary Material 1

Supplementary Material 2

## Data Availability

All data relating to the present study are available in this manuscript, Table S1, and Table S2 files.
