# Supplementary material for: A systematic review of circulating IP-10/CXCL10 in patients with Plasmodium infections in relation to disease severity
Source: Sci Rep. 2024 Dec 30;14:31723. doi: 10.1038/s41598-024-82712-0 (PMC11685427; doi:10.1038/s41598-024-82712-0)
Supplement: Supplementary file 1 — Supplementary Material 1 [file 41598_2024_82712_MOESM1_ESM.docx]

**Table S1. Search terms**

**General keywords**

(“interferon gamma-induced protein-10” OR IP-10 OR CXCL10 OR “Interferon-Inducible Protein 10” OR “Interferon Inducible Protein 10” OR “Small Inducible Cytokine B10” OR “IFN-gamma-Inducible Protein” OR “CXCL10 Chemokine” OR “gammaIP-10 Protein” OR “gammaIP 10 Protein” OR “Chemokine (C-X-C Motif) Ligand 10”) AND (malaria OR plasmodium OR “Plasmodium Infection“ OR “Remittent Fever“ OR “Marsh Fever“ OR Paludism)

PubMed 23 April 2024

| No. | Key concept | Search terms | Results |
| --- | --- | --- | --- |
| 1. | IP-10 | “interferon gamma-induced protein-10”[All Fields] OR IP-10[All Fields] OR CXCL10[All Fields] OR “Interferon-Inducible Protein 10”[All Fields] OR “Interferon Inducible Protein 10”[All Fields] OR “Small Inducible Cytokine B10”[All Fields] OR “IFN-gamma-Inducible Protein”[All Fields] OR “CXCL10 Chemokine” [All Fields] OR “gammaIP-10 Protein”[All Fields] OR “gammaIP 10 Protein”[All Fields] OR “Chemokine (C-X-C Motif) Ligand 10”[All Fields] OR “interferon gamma-induced protein-10”[MeSH Terms] OR IP-10[MeSH Terms] OR CXCL10[MeSH Terms] | 12,732 |
| 2. | Malaria | "malaria"[MeSH Terms] OR "malaria"[All Fields] OR "malarias"[All Fields] OR "malaria s"[All Fields] OR "malariae"[All Fields] OR ("plasmodium"[MeSH Terms] OR "plasmodium"[All Fields] OR "plasmodiums"[All Fields] OR "plasmodium s"[All Fields]) OR "Plasmodium Infection"[All Fields] OR "Remittent Fever"[All Fields] OR "Marsh Fever"[All Fields] OR ("malaria"[MeSH Terms] OR "malaria"[All Fields] OR "paludism"[All Fields]) | 126,567 |
| 3. | #1 AND #2 | (“interferon gamma-induced protein-10”[All Fields] OR IP-10[All Fields] OR CXCL10[All Fields] OR “Interferon-Inducible Protein 10”[All Fields] OR “Interferon Inducible Protein 10”[All Fields] OR “Small Inducible Cytokine B10”[All Fields] OR “IFN-gamma-Inducible Protein”[All Fields] OR “CXCL10 Chemokine” [All Fields] OR “gammaIP-10 Protein”[All Fields] OR “gammaIP 10 Protein”[All Fields] OR “Chemokine (C-X-C Motif) Ligand 10”[All Fields] OR “interferon gamma-induced protein-10”[MeSH Terms] OR IP-10[MeSH Terms] OR CXCL10[MeSH Terms]) AND ("malaria"[MeSH Terms] OR "malaria"[All Fields] OR "malarias"[All Fields] OR "malaria s"[All Fields] OR "malariae"[All Fields] OR ("plasmodium"[MeSH Terms] OR "plasmodium"[All Fields] OR "plasmodiums"[All Fields] OR "plasmodium s"[All Fields]) OR "Plasmodium Infection"[All Fields] OR "Remittent Fever"[All Fields] OR "Marsh Fever"[All Fields] OR ("malaria"[MeSH Terms] OR "malaria"[All Fields] OR "paludism"[All Fields])) | 129 |

Embase 23 April 2024

| No. | Key concept | Search terms | Results |
| --- | --- | --- | --- |
| 1. | IP-10 | “interferon gamma-induced protein-10”:ti,ab,kw,de OR IP-10:ti,ab,kw,de OR CXCL10:ti,ab,kw,de OR “Interferon-Inducible Protein 10”:ti,ab,kw,de OR “Interferon Inducible Protein 10”:ti,ab,kw,de OR “Small Inducible Cytokine B10”:ti,ab,kw,de OR “IFN-gamma-Inducible Protein”:ti,ab,kw,de OR “CXCL10 Chemokine”:ti,ab,kw,de OR “gammaIP-10 Protein”:ti,ab,kw,de OR “gammaIP 10 Protein”:ti,ab,kw,de OR “Chemokine (C-X-C Motif) Ligand 10”:ti,ab,kw,de OR “interferon gamma-induced protein-10”/exp OR IP-10/exp OR CXCL10/exp | 32,949 |
| 2. | Malaria | malaria:ti,ab,kw,de OR plasmodium:ti,ab,kw,de OR ‘Remittent Fever’:ti,ab,kw,de OR ‘Marsh Fever’:ti,ab,kw,de OR Paludism:ti,ab,kw,de OR malaria/exp | 161,531 |
| 3. | 1 AND 2 | (“interferon gamma-induced protein-10”:ti,ab,kw,de OR IP-10:ti,ab,kw,de OR CXCL10:ti,ab,kw,de OR “Interferon-Inducible Protein 10”:ti,ab,kw,de OR “Interferon Inducible Protein 10”:ti,ab,kw,de OR “Small Inducible Cytokine B10”:ti,ab,kw,de OR “IFN-gamma-Inducible Protein”:ti,ab,kw,de OR “CXCL10 Chemokine”:ti,ab,kw,de OR “gammaIP-10 Protein”:ti,ab,kw,de OR “gammaIP 10 Protein”:ti,ab,kw,de OR “Chemokine (C-X-C Motif) Ligand 10”:ti,ab,kw,de OR “interferon gamma-induced protein-10”/exp OR IP-10/exp OR CXCL10/exp) AND (malaria:ti,ab,kw,de OR plasmodium:ti,ab,kw,de OR ‘Remittent Fever’:ti,ab,kw,de OR ‘Marsh Fever’:ti,ab,kw,de OR Paludism:ti,ab,kw,de OR malaria/exp) | 321 |

Scopus 23 April 2024

| No. | Key concept | Search terms | Results |
| --- | --- | --- | --- |
| 1. | IP-10 | TITLE-ABS-KEY (“interferon gamma-induced protein-10” OR IP-10 OR CXCL10 OR “Interferon-Inducible Protein 10” OR “Interferon Inducible Protein 10” OR “Small Inducible Cytokine B10” OR “IFN-gamma-Inducible Protein” OR “CXCL10 Chemokine” OR “gammaIP-10 Protein” OR “gammaIP 10 Protein” OR “Chemokine (C-X-C Motif) Ligand 10”) | 24,533 |
| 2. | Malaria | TITLE-ABS-KEY (malaria OR plasmodium OR "plasmodium infection" OR "remittent fever" OR "marsh fever" OR paludism) | 162,821 |
| 3. | 1 AND 2 | ( TITLE-ABS-KEY ( "interferon gamma-induced protein-10" OR ip-10 OR cxcl10 OR "Interferon-Inducible Protein 10" OR "Interferon Inducible Protein 10" OR "Small Inducible Cytokine B10" OR "IFN-gamma-Inducible Protein" OR "CXCL10 Chemokine" OR "gammaIP-10 Protein" OR "gammaIP 10 Protein" OR "Chemokine (C-X-C Motif) Ligand 10" ) ) AND ( TITLE-ABS-KEY ( malaria OR plasmodium OR "plasmodium infection" OR "remittent fever" OR "marsh fever" OR paludism ) ) | 275 |

MEDLINE 23 April 2024

| No. | Key concept | Search terms | Results |
| --- | --- | --- | --- |
| 1. | IP-10 AND Malaria | (“interferon gamma-induced protein-10” OR IP-10 OR CXCL10 OR “Interferon-Inducible Protein 10” OR “Interferon Inducible Protein 10” OR “Small Inducible Cytokine B10” OR “IFN-gamma-Inducible Protein” OR “CXCL10 Chemokine” OR “gammaIP-10 Protein” OR “gammaIP 10 Protein” OR “Chemokine (C-X-C Motif) Ligand 10”) AND (malaria OR plasmodium OR “Plasmodium Infection“ OR “Remittent Fever“ OR “Marsh Fever“ OR Paludism) | 123 |

Ovid 23 April 2024

| No. | Key concept | Search terms | Results |
| --- | --- | --- | --- |
| 1. | IP-10 AND Malaria | ("interferon gamma-induced protein-10" OR IP-10 OR CXCL10 OR "Interferon-Inducible Protein 10" OR "Interferon Inducible Protein 10" OR "Small Inducible Cytokine B10" OR "IFN-gamma-Inducible Protein" OR "CXCL10 Chemokine" OR "gammaIP-10 Protein" OR "gammaIP 10 Protein" OR "Chemokine (C-X-C Motif) Ligand 10") AND (malaria OR plasmodium OR "Plasmodium Infection" OR "Remittent Fever" OR "Marsh Fever" OR Paludism) {Including Limited Related Terms} | 622 |

ProQuest 23 April 2024

| No. | Key concept | Search terms | Results |
| --- | --- | --- | --- |
| 1. | IP-10 AND Malaria | ("interferon gamma-induced protein-10" OR IP-10 OR CXCL10 OR "Interferon-Inducible Protein 10" OR "Interferon Inducible Protein 10" OR "Small Inducible Cytokine B10" OR "IFN-gamma-Inducible Protein" OR "CXCL10 Chemokine" OR "gammaIP-10 Protein" OR "gammaIP 10 Protein" OR "Chemokine (C-X-C Motif) Ligand 10") AND (malaria OR plasmodium OR "Plasmodium Infection" OR "Remittent Fever" OR "Marsh Fever" OR Paludism) | 463 |

Google Scholar 24 April 2024

| No. | Key concept | Search terms | Results |
| --- | --- | --- | --- |
| 1. | IP-10 AND Malaria | (IP-10 OR CXCL10) AND malaria | The first 200 articles |
